# Mother's Knowledge on HIV, Syphilis, Rubella, and Associated Factors in Northern Tanzania: Implications for MTCT Elimination Strategies

**DOI:** 10.1155/2020/7546954

**Published:** 2020-07-07

**Authors:** Nikolas A. S. Chotta, Sia E. Msuya, Melina Mgongo, Tamara H. Hashim, Arne Stray-Pedersen

**Affiliations:** ^1^Institute of Clinical Medicine, University of Oslo, Norway; ^2^Better Health for African Mother and Child (BHAMC), Moshi, Tanzania; ^3^Department of Community Health, Institute of Public Health, Kilimanjaro Christian Medical University College (KCMUCo), Moshi, Tanzania; ^4^Department of Epidemiology and Biostatistics, Institute of Public Health, Kilimanjaro Christian Medical University College (KCMUCo), Moshi, Tanzania; ^5^Institute of Basic Sciences, University of Oslo, Norway; ^6^Department of Forensic Medicine, Oslo University Hospital, Oslo, Norway

## Abstract

**Background:**

Infections transmitted from mother to child (MTCT) during pregnancy, childbirth, and breastfeeding contribute significantly to the high infant and childhood morbidity and mortality in sub-Saharan African countries. The most significant and preventable of these include HIV, syphilis, and rubella. To achieve elimination, mothers need to be aware of and to understand effective preventive measures against these infections. Lack of comprehensive knowledge on transmission and prevention of MTCT infections is one of the factors hindering achievement of the elimination goals for these infections. The aim of this study was to assess the knowledge of HIV, syphilis, rubella, and associated factors among mothers in the Kilimanjaro region of Tanzania.

**Methods:**

We conducted a community-based cross-sectional study in three districts of the Kilimanjaro region from September to October 2016. The study involved mothers with children up to five years of age. Data collection involved the use of a questionnaire, administered by face-to-face interviews. Logistic regression analysis was used to assess predictors of mothers' knowledge on MTCT infections.

**Results:**

A total of 618 mothers were recruited, with a mean age of 29.6 (SD 7.6) years. The overall knowledge on MTCT infections was low. The highest level of knowledge on MTCT infections was regarding HIV (89.2%). Fewer mothers had knowledge of syphilis (27.8%). Rubella was the least known; only 12% of mothers were aware of rubella infection. District of residence and having knowledge of syphilis were predictors for rubella knowledge, while for syphilis knowledge, significant predictors were age group, occupation, and those having knowledge on HIV and rubella. Predictors for HIV knowledge were residential district, having a mobile phone, and those having knowledge of syphilis and rubella.

**Conclusions:**

This study confirmed that mothers have low overall knowledge on MTCT infections. To achieve the MTCT elimination goals, targeted interventions to improve knowledge among women of childbearing age are recommended.

## 1. Background

Paediatric human immunodeficiency virus (HIV), congenital syphilis, and congenital rubella syndrome are still of public health importance in developing countries, including Tanzania [1, 2]. The three infections, HIV, syphilis, and rubella, can be transmitted from the mother to the child (MTCT) during pregnancy, during birth (HIV and syphilis), and during breastfeeding (HIV). The three MTCT infections contribute to a significant proportion of child morbidity and mortality. At the end of 2017, there were 1.8 million children living with HIV globally. In the same year, there were 180,000 new HIV infections among children, mostly acquired through mother-to-child transmission (MTCT). An estimate of 110,000 children died from lack of treatment [3]. Most of these HIV infections and deaths happened in sub-Saharan Africa (SSA) including Tanzania [3, 4]. In the same year, more than 500,000 cases of congenital syphilis (CS) were reported, which resulted in over 515,000 premature births, low birth weight (LBW), various manifestations of inflammation, and neonatal deaths [5, 6]. Premature births and/or LBW is the second leading cause of neonatal deaths in Tanzania.

Congenital rubella syndrome (CRS) results from primary rubella infection in the first trimester of pregnancy and causes severe birth defects in the eye, ear, heart, spleen, liver, and brain. The global incidence is 0.1 to 0.2 per 1000 live births. Globally in 2017, there were 22,361 reported cases of congenital rubella syndrome [7, 8].

Primary prevention, screening and treatment for HIV and syphilis, and vaccination for rubella are established services for the prevention of mother-to-child transmission (PMTCT). The World Health Organization (WHO) advocates for global elimination of these three MTCT infections using available health services. Despite the availability of preventive services, goals for elimination of MTCT infections are not being reached in most SSA countries [4]. Factors which influence elimination efforts, such as health care system bottlenecks for HIV, syphilis, and rubella screening, treatment, and surveillance, have been described before [4, 9]. There is limited information on client factors which influence progress towards elimination goals for MTCT infections [10–14].

Inadequate knowledge on MTCT infections has been cited as one of the barriers to attaining elimination goals [4]. The four prongs of the PMTCT approach target the four periods of MTCT; before conception, throughout pregnancy, during delivery, and during breastfeeding [4, 15–18]. Women with low knowledge on PMTCT have lower acceptance and adherence to PMTCT services [11, 15–18]. Pregnant women and women of reproductive age, who are key stakeholders in MTCT elimination, need adequate knowledge of the risks and available preventive measures. There is documented low knowledge on PMTCT among women in African countries, where the burden of MTCT of infection is highest [15–21].

In Kenya, a study by Thomson et al. revealed that among HIV-infected pregnant women enrolled in a free antiretroviral treatment (ART) in Nairobi, the attrition rate was up to 43% within the first year of treatment. Lack of knowledge on HIV and PMTCT of HIV were among the main factors for the high dropout rates [22]. In a study of women from Ghana on antiretroviral treatment (ART), 90% of defaulters had inadequate knowledge of ART and PMTCT [17]. Similarly, in Ethiopia, a study by Abtew et al. found that at least half of pregnant women had good knowledge of MTCT of HIV; however, less than a quarter had comprehensive knowledge of PMTCT of HIV [18]. In Zambia, a study among women on ART from pregnancy to a six-month postnatal period found increasing suboptimal adherence to services over time. Poor knowledge was one of the contributing risk factors [19]. In Tanzania, lack of comprehensive knowledge on PMTCT of HIV has been associated with increased stigma, nondisclosure, poor adherence to ART, defaulting, and switching to alternative therapies [21, 23–25]. In all the SSA countries, there is dearth of information about women's knowledge and practice of other MTCT transmissible infections like syphilis and rubella.

Tanzania has adapted most of the evidence-based interventions to reduce MTCT of transmissible infections. It has in place a national program for no-cost HIV and syphilis screening and management since early 2000 [26], as well as a program for a paediatric follow-up and care for HIV-exposed and HIV-infected children [23, 26]. In 2014, the country introduced a vaccine for rubella [27]. It is given as a combination measles and rubella (MR) vaccine to children at 9 and 18 months of age. Thus, the elimination of CRS depends on sustained high MR vaccine coverage, which requires adequate knowledge among mothers for vaccine acceptance [28]. However, despite these intervention programs, there is still a high risk of MTCT infections. The national rate of MTCT for HIV is 6-11% [29, 30]. More than 160,000 children are infected with HIV in the country. In the year 2018, there were additional 8600 HIV-infected children. HIV/AIDS contributed to 18% of childhood mortality [23]. The risk for MTCT of HIV is high due to a low testing rate and high HIV prevalence among reproductive age women which are at 60% and 6.2%, respectively [23, 25, 31, 32]. Furthermore, among pregnant women, HIV prevalence is high at 6.5%; more than half of pregnant mothers have late initiation of ARVs and 9% are not tested for HIV [23, 25]. The burden of syphilis in pregnancy is still high in Tanzania, and every year, more than 100,000 pregnant women are infected with syphilis (6.9%). More than 51% of stillbirths are attributed to syphilis [12, 14, 33]. Antenatal screening for syphilis is very low (30-38%) leading to a high risk for congenital syphilis [33, 34]. National data on congenital syphilis are scanty [12, 14, 33]. Few studies have documented the burden and risk of MTCT of rubella infection in Tanzania. Among pregnant women, 2.7 to 12% of adverse pregnancy outcomes are attributed to acute rubella infection [35, 36]. Risk factors for CRS are low herd immunity, young mothers, lack of rubella seroprevalence screening, not vaccinating all school girls, and low rubella vaccine coverage [37, 38]. In the study region, Kilimanjaro, the population HIV prevalence is below the national average [14, 25, 32, 33]. PMTCT for HIV services are well integrated in the antenatal clinics, attendance for one antenatal care visit is close to 100%, and a high proportion of pregnant women are tested for HIV [14, 25]. However, antenatal syphilis screening is low and the susceptibility to rubella is high [14, 33, 36]. The persisting high-burden MTCT of HIV, CS, and CRS in developing countries like Tanzania calls for an urgent need to assess levels and factors that affect the application of prevention programs [9].

This study therefore is aimed at describing (i) women's awareness and knowledge of MTCT infections in communities in the Kilimanjaro region, northern Tanzania, and (ii) the determinant factors for knowledge of MTCT infections at the individual level.

Most of the previous studies on knowledge of PMTCT were limited to HIV and did not address the other infections in the elimination agenda [15, 16, 39, 40]. The information from this study will contribute to the development of effective anticipatory guidance to mothers and their families. The findings will also complement existing strategies for the prevention and achievement of elimination goals for MTCT of HIV, syphilis, and rubella.

## 2. Materials and Methods

### 2.1. Study Design and Area

This was a community-based cross-sectional study which was conducted from September to October 2016. The study was conducted in three districts of the Kilimanjaro region in northeastern Tanzania. The districts included in the study were Moshi Municipality, Rombo, and Same. The Kilimanjaro region has a literacy rate above 92%, a high engagement with reproductive health services (antenatal attendance of 99% vs. the national average of 94%; a high rate of women giving birth in health facilities (96% vs. 64%), and a high vaccination coverage rate—for infants at four weeks, it is at 100%. The general childhood vaccination rate is 93% vs. the national average of 75% [25, 41].

### 2.2. Study Population

The study population included mothers with children below 5 years of age residing in the 3 selected districts of the Kilimanjaro region. The inclusion criteria included biological mothers who gave informed consent to participate, permanent residents in the selected districts, individuals aged 18 years and above, and mothers with living children up to five years of age.

### 2.3. Calculation of the Sample Size

The estimated sample size was calculated by using the sample size formula for cross-sectional studies: *N* = *Z*^2^*p* (1 − *p*)/*d*^2^, where *N* is the estimated minimum sample size, *Z* is the confidence level of 95% (the standard value is 1.96), *P* is the expected proportion of women with knowledge of MTCT routes for HIV in the population = 50% (based on previous studies in the area, [15, 25], and *d* is the precision at 95%CI = 0.05.

With an adjustment of 20% for nonresponse, a minimum sample size of 460 women with children below 5 years was required for the study.

### 2.4. Sampling Methods

A multistage sampling technique was used to select urban and rural representative populations as follows:

*Stage 1*. Selection of the districts to participate in the study was conducted. The seven districts in the Kilimanjaro region were categorized into three predominantly urban (Moshi Municipality, Moshi District, and Hai Councils) and four predominantly rural districts (Rombo, Mwanga, Same, and Siha). One urban (Moshi Municipality) and two rural districts (Rombo and Same) were randomly selected.

*Stage 2*. This stage involved the selection of the wards in each of the three districts. In Moshi Municipality, 5 out of 21 wards were randomly selected to participate, using a ballot method. The five wards from the urban district were Bondeni, Korongoni, Majengo, Pasua, and Kiboriloni. Eleven out of 55 wards in the rural districts were randomly selected. The wards were Vumari, Mwembe, Stesheni, Ndungu, Makanya, Tarakea, Motamburu, Mamsera, Kelamfua Mokala, Katangare, Kirwa Keni, and Mrao Keryo.

*Stage 3*. The 3^rd^ stage involved the selection of streets in urban and villages in the rural districts. From the selected wards, all the villages/streets were listed by simple random sampling. Fifteen streets from Moshi Municipality and 61 villages in Same and Rombo districts were selected. The village executive officers and hamlet leaders in the selected 78 villages/streets were involved in identifying all resident mothers with children up to five years who met the inclusion criteria to be included in the study.

### 2.5. Data Collection Procedures

Data collection was conducted by senior medical students who were trained and supervised by three researchers. In the selected villages and streets, all mothers with children up to five years were visited in their households. For those meeting the inclusion criteria, after obtaining informed written consent, data was collected by face-to-face interviews. A questionnaire was used for data collection. The following information was collected during the interviews: sociodemographic data, reproductive health history, the source of their information about reproductive and child health services, and their knowledge of MTCT infections (HIV, syphilis, rubella, and other infections; risk factors; transmission methods; consequences and preventive methods).

### 2.6. Data Management and Analysis

The filled questionnaires were checked for completeness at the end of each working day. The collected data was coded and entered into the computer software. Research subjects were revisited in cases of missing data. Statistical analysis was performed using the Statistical Package for the Social Sciences (SPSS) for Windows version 22. Knowledge on MTCT infections was the main dependent variable and was defined as the correct response to the following questions. (i) The MTCT infections, (ii) the mode of transmission for the mentioned MTCT infection, (iii) the consequences of the MTCT infection, and (iv) how to prevent the mentioned MTCT infection are correctly identified. The sociodemographics—age, sex, marital status, education, occupation, socioeconomic status, family size, household utilities, reproductive health history, source of information for reproductive and child health (RCH) services—were the main independent variables. Descriptive statistics were used to summarize the data. Categorical data were summarized by frequencies and proportions, and means and standard deviations were calculated for continuous variables. Odds ratios and their 95% confidence intervals were calculated to test for associations between dependent and independent variables.

### 2.7. Ethical Considerations

The study was approved by the Kilimanjaro Christian Medical University College Research Ethical Committee (CREC: certificate number 917). Since there were no human tissue sample collection and testing, an exemption for ethical approval was granted by the Regional Ethics Committee (REK) in South-East Norway.

Written permission was sought from the respective District Executive Directors.

Written informed consent was obtained from each participating mother prior to the interview. Numbers were used on questionnaires to protect the participants' identities. All information obtained was secured by the principal investigator.

## 3. Results

### 3.1. Background Characteristics of Participants

The study recruited 618 mothers from 78 streets/villages in the three districts of the Kilimanjaro region. The mean age of the 618 mothers was 29.6 years (standard deviation (SD) = 7.6 years). A high proportion of mothers were married or cohabiting (76.0%), had completed primary education (70.1%), and had access to piped water (89%). The majority of the mothers were doing small-scale businesses or farming (82.9%). More than half had electricity in their homes (54.4%) and used pit latrines (52.6%) (Table 1).

Of the 618 mothers, 99.7% reported that they received antenatal care (ANC) at least once in their last pregnancy and about three quarters (77.6%) received care four or more times. Reported delivery at a medical facility was high (95.0%).

A total of 89% of mothers reported screening for HIV in their last pregnancy, while this proportion was 13.4% for syphilis (Table 2).

### 3.2. Knowledge of MTCT Infections

The majority of participating mothers (89.2%) demonstrated knowledge of HIV as an MTCT infection. A small proportion knew about syphilis (27.8%). On direct probing on the consequences of syphilis infection during pregnancy, 28.8% of the mothers had correct responses. Of the 178 who knew syphilis complications during pregnancy, 15.7% mentioned miscarriage, 7.8% mentioned stillbirths, and only 2.1% mentioned congenital syphilis as a consequence (Table 3).

**Figure 1:**
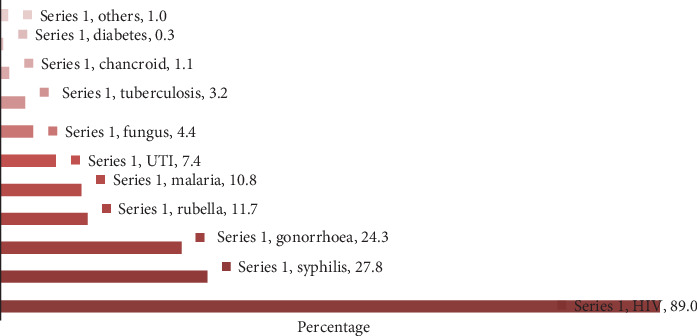
Conditions and/or infections mentioned by women that can be transmitted from mother to child in Kilimanjaro (*N* = 618).

Women also mentioned other conditions or infections they wrongly considered to be transmitted from mothers to children, e.g., urinary tract infection (UTI), fungus, tuberculosis, and diabetes (Figure 1).

The overall knowledge of rubella was low; a higher proportion of mothers did not know about rubella infection (88.3%). Almost none knew how rubella is spread (99.0%), the consequences of primary rubella infection during early pregnancy (99.7%), or how to prevent rubella infection (99.7%).

### 3.3. Factors Associated with Knowledge of MTCT of HIV, Syphilis, and Rubella

Table 4 shows the association between background factors and knowledge of MTCT of HIV, syphilis, and rubella.

Table 5 shows the independent predictors for a mother's knowledge on the three MTCT infections.

## 4. Discussion

To our knowledge, this is the first study describing awareness and knowledge of MTCT infections after the introduction of the rubella vaccine in Tanzania. Although HIV was the most known infection at 89.0%, it is concerning to observe that despite 99% of the women receiving ANC at least once and 78% receiving ANC 4 or more times, 10% and 70% of mothers were not aware of HIV and syphilis as MTCT infections, respectively. Several studies have reported that in resource-limited settings, despite participation in antenatal care, there is a failure to impart comprehensive knowledge to attendees, resulting in poor comprehension and retention in the PMTCT cascade [12, 13, 42, 43].

The mother's HIV knowledge level in this study (89%) was higher than levels observed in a previous study by Falnes et al. in Moshi which reported knowledge on MTCT of HIV at 70.5% [25]. The increased knowledge in our study may be attributed to maturation of the PMTCT program over the years and the adopted multisectoral approach in combating HIV since 2000 [26]. While there is an improvement, the remaining 11% of women unaware of HIV are worrying given the technical and financial investments in interventions for MTCT of HIV in the country. Lack of awareness and knowledge results in poor adherence and outcomes in PMTCT interventions, stigma, lack of disclosure, loss of follow-up, and increased MTCT of infections. This accumulates in an ultimate denial of empowerment of women to protect their children [6, 10, 17, 18, 26]. There is a need to improve anticipatory guidance on MTCT infections in order to improve maternal adherence to prevention and recognition guidelines for reduction in newborn and child morbidity and mortality.

The low awareness of 27.8% and screening for syphilis have been reported in several studies and are mainly due to health care system bottlenecks and socioeconomic factors [25]. While HIV prevention is well integrated in antenatal care and there is a multisectoral response against it, for syphilis, there is a limited scope in prevention campaigns, despite its high prevalence and the severe consequences on pregnancy and fetal outcomes [26]. In response to this and in an effort to achieve the dual elimination goal, the WHO recommends the use of dual rapid tests for both HIV and syphilis. This should bridge the gap and increase both knowledge and screening for syphilis during antenatal care [10]. Strengthening of universal antenatal syphilis screening is urgently needed in this setting and Tanzania as a whole in order to eliminate morbidity and mortality from congenital syphilis [12–14].

Rubella was the least known MTCT infection among interviewed mothers, with only one in ten who had ever heard about rubella. Despite the countrywide campaigns for the rubella vaccination since 2014 [27], lack of rubella knowledge among health care workers partly explains this trend. In a previous study in the Kilimanjaro region, we found that health care workers had poor knowledge on rubella at 26% [44]. The health care workers remain the most trusted source for health information. This may explain why rubella is little known, despite its severe consequences on pregnancy and subsequent designation as a targeted disease for elimination through routine vaccination in Tanzania [27, 42–44]. The low knowledge on rubella in our study is different to the findings in a study by Morioka et al. among pregnant women in Japan, where rubella was the second most known MTCT infection after HIV. 76% of pregnant women were knowledgeable regarding rubella [45]. The contrast may be due to differences in resources for health, as well as program maturation which are favourable for Japan, which is a high-income country. Japan has had a rubella vaccination program since 1986, while Tanzania, a low-income country, has only recently introduced the rubella vaccine, in 2014 [27, 45].

Low knowledge on rubella among women of reproductive age has been linked with low acceptance of rubella vaccination. In a study of children in Mtwara, Tanzania, by Magodi et al., less than half of eligible children had received the second measles-rubella (MR2) vaccine. Among the main factors for this low coverage was lack of knowledge on the vaccination schedule among caretakers [46]. Similarly, another study in Ethiopia by Negussie et al. found that lack of knowledge of the effectiveness of vaccines was one of the reasons for noncompliance of scheduled vaccines [46]. Tanzania is lagging behind on its MR2 coverage [37]. Despite the high first (MR1) coverage of >98%, the repeat vaccine (MR2) coverage is at 84%, below the national target of 90% [38]. Wide regional variations in MR vaccine coverage illustrate the need for studies to explore the contributing factors [37]. There is an urgent need for integrated campaigns to target all preventable MTCT infections, including creating awareness of CRS prevention by MR vaccination. Increasing parental knowledge of rubella will increase compliance with the second MR dose and supplemental vaccines for CRS elimination [28].

The study noted existing misconceptions on the diseases with possible MTCT. Mothers mentioned some conditions that have an impact on pregnancy but are not MTCT infections, such as diabetes mellitus. In Tanzania, though health care workers routinely screen and give anticipatory guidance for common problems and danger signs in pregnancy, the quality of counselling in those encounters is inadequate. This may explain the low awareness and lack of comprehensive knowledge, leading to the reported misconceptions [42]. These findings are reported in several studies in SSA countries [15–21].

In this study, the district of residence was a predictor of rubella and HIV knowledge. Compared to Rombo District, women in Moshi urban and Same had less knowledge of HIV. This finding of higher levels of knowledge in a predominantly rural district differs from other studies where rural areas were found to have lower knowledge [18, 20]. Knowledge of one of the MTCT infections increased the odds of having knowledge of the other two of the studied mother-to-child transmissible infections. The study found that having knowledge of syphilis was a predictor for having knowledge of rubella. Also, having rubella and syphilis knowledge was an independent predictor for knowledge of HIV as an MTCT infection. This emphasises that it is easier for women to grasp and retain knowledge if the information is given in a grouped and integrated manner, rather than delivering health education for each single infection [26, 28, 42].

Mothers' knowledge on syphilis increased with age and among those with formal employment. This finding is reported by other antenatal syphilis studies. Manyahi et al., in a study among antenatal mothers in Tanzania, reported that the risk of syphilis was highest among multiparous women with 2-5 pregnancies and those living in rural areas. The increased risk and cumulative encounters with health care workers may explain the increased knowledge with advancing age and parity [33]. Mothers with formal occupations may be more empowered by education, social interactions, access to media, good economic status, and better access to health services. This may explain the high knowledge on syphilis among mothers with formal employment [40].

The study had several strengths. It was a community-based study on randomly selected women and districts in the Kilimanjaro region. The findings can therefore be generalized for the region. The study has weaknesses as well that must be considered. We enrolled women with living under-five children, who provided self-reports; hence, it has both survival and recall bias. Despite the inherent limitations, the study presents useful information of the complex interaction of factors in this community and the challenges in eliminating the three MTCT infections.

## 5. Conclusion and Recommendations

The mother's knowledge of mother-to-child transmissible infections was low for syphilis and rubella. HIV was the most known MTCT infection, and consequently, most mothers had been tested for HIV. The high health service utilization is an opportunity to improve education and screening for other MTCT infections, e.g., by linking HIV and syphilis rapid tests and increasing MR vaccine coverage.

To attain the elimination goals, improved public health education of MTCT infection is required. We also recommend increasing the awareness and accessibility of screening and prevention for MTCT infections to all people of reproductive age.

## Figures and Tables

**Table 1:** Baseline characteristics of the participants (*N* = 618).

Variable	Frequency	%
District of enrolment		
Moshi Municipality	159	25.7
Same	244	39.5
Rombo	215	34.8
Age group (years)		
15-24	190	30.7
25-34	258	41.7
35-44	153	24.8
45-55	17	2.8
Marital status		
Married	413	66.8
Cohabiting	57	9.2
Single	113	18.3
Widow/divorced/separated	35	5.7
Level of education		
None	11	1.8
Primary	433	70.1
Secondary or higher	174	28.1
Women's occupation		
Formally employed	32	5.2
Subsistence farmer	282	45.7
Small business	230	37.2
Housewife/none	74	12
Ownership of the house they live in		
Own the house	223	36.1
Renting	212	35.8
Family house	174	28.2
Have the following at home (yes)		
Electricity	336	54.4
Television	222	36.8
Radio	399	65.8
Mobile phone	539	87.6
Water source at household		
Piped water	552	89.3
Well/spring/dam	66	10.7
Type of toilet at household		
Pit latrine	325	52.6
Flush toilet	293	47.4
Occupation of the partner (*N* = 546)		
Employed	63	11.5
Self-employed	201	36.8
Subsistence farmer	189	34.6
Others	93	17.0

**Table 2:** Reproductive and maternal history among 618 women in the Kilimanjaro region.

Variable	Frequency	%
Attended ANC at the last pregnancy		
No	2	0.3
Yes	616	99.7
Frequency of ANC attendance (last pregnancy)		
Once	24	3.9
2-3 times	114	18.5
4 or more	478	77.6
Did the mother receive MTCT infection screening during pregnancy?		
No	16	2.6
Yes	602	97.4
Did the partner come for HIV screening during pregnancy?		
No	275	44.5
Yes	343	55.5
MTCT infection screening during pregnancy		
HIV	552	89.3
Syphilis	83	13.4
MTCT infections diagnosed during pregnancy screening		
HIV (*N* = 552)	14	2.5
Syphilis (*N* = 83)	3	3.6
Place of delivery for the youngest child		
Home delivery	31	5.0
Facility delivery	587	95.0

**Table 3:** Knowledge of MTCT infections among 618 women in the Kilimanjaro region.

Variable	Frequency	%
Mentioned HIV and/or syphilis as mother-to-child transmissible infections		
None	66	10.7
One	386	62.5
Both	166	26.9
Mentioned HIV as a mother-to-child transmissible infection		
No	68	11.8
Yes	550	89.2
Mentioned syphilis as a mother-to-child transmissible infection		
No	446	72.2
Yes	172	27.8
Consequence of syphilis in pregnancy		
Know consequence of syphilis in pregnancy		
No	440	71.2
Yes	178	28.8
Mentioned miscarriage as a consequence of syphilis in pregnancy		
No	521	84.3
Yes	97	15.7
Mentioned stillbirth as a consequence of syphilis in pregnancy		
No	570	92.2
Yes	48	7.8
Mentioned congenital syphilis/deformity as a consequence of syphilis in pregnancy		
No	605	97.9
Yes	13	2.1
Rubella awareness and consequence in pregnancy		
Ever heard of rubella		
No	546	88.3
Yes	72	11.7
Can explain how rubella is spread		
No	612	99.0
Yes	6	1.0
Know the consequence of rubella in pregnancy		
No	615	99.7
Yes	2	0.3
Know rubella can be prevented with vaccination		
No	615	99.7
Yes	2	0.3

**Table 4:** Factors associated with knowledge of rubella, syphilis, and HIV as MTCT infections (*N* = 618).

Variable	*N*	Rubella			Syphilis			HIV		
		Knowledge (*n* (%))	COR (95% CI)	*P* value	Knowledge (*n* (%))	COR (95% CI)	*P* value	Knowledge (*n* (%))	COR (95% CI)	*P* value
District of enrollment										
Rombo	215	18 (8.4)	1		60 (27.9)	1		198 (92.1)	1	
Moshi urban	159	16 (10.1)	1.23 (0.61-2.50)	0.561	48 (30.2)	1.12 (0.71-1.75)	0.63	135 (84.9)	0.48 (0.25-0.93)	0.03
Same	244	38 (15.6)	2.02 (1.11-3.66)	0.02	62 (25.4)	0.88 (0.58-1.33)	0.546	212 (86.9)	0.57 (0.31-1.06)	0.074
Age group (years)										
15-24	190	22 (11.6)	1		32 (16.8)	1		160 (84.2)	1	
25-34	258	32 (12.4)	1.07 (0.61-1.92)	0.807	72 (27.9)	1.91 (1.20-3.05)	0.007	235 (91.1)	1.92 (1.07-3.42)	0.028
35+	170	18 (10.6)	0.90 (0.46-1.74)	0.752	66 (38.8)	3.13 (1.92-5.11)	<0.001	150 (88.2)	1.41 (0.77-2.58)	0.272
Marital status										
Married/cohabiting	470	54 (11.5)	1.0		139 (29.6)	1.0		420 (89.4)	1.0	
Single	113	17 (15.0)	1.36 (0.76-2.46)	0.305	20 (17.7)	0.51 (0.30-0.86)	0.012	92 (81.4)	0.52 (0.30-0.91)	0.022
Widow/div/separated	35	1 (2.9)	0.23 (0.03-1.69)	0.147	11 (31.4)	1.09 (0.52-2.29)	0.817	33 (94.3)	1.96 (0.46-8.43)	0.364
Level of education										
None/primary	444	46 (10.4)	1.0		124 (27.9)	1.0		386 (86.9)	1.0	
Secondary or higher	174	26 (15.0)	1.53 (0.91-2.57)	0.124	46 (26.4)	0.93 (0.62-1.38)	0.764	159 (91.4)	1.59 (0.88-2.89)	0.13
Women's occupation										
Not formally employed	586	67 (11.5)	1		154 (26.3)	1.0		516 (88.1)	1.0	
Formally employed	32	5 (15.6)	1.43 (0.53-3.84)	0.406	16 (50.0)	1.90 (1.31-2.76)	0.005	29 (90.6)	1.31 (0.39-4.42)	0.662
Ownership of radio										
No	207	31 (15.0)	1.0		56 (27.1)	1.0		177 (85.5)	1.0	
Yes	399	39 (9.8)	0.99 (0.47-2.08)	0.062	110 (27.6)	1.02 (0.77-1.34)	0.924	358 (89.7)	1.05 (0.98-1.12)	0.143
Have mobile phone										
No	76	9 (11.8)	1.0		15 (19.7)	1.0		59 (77.6)	1.0	
Yes	539	63 (11.7)	0.99 (0.47-2.08)	0.973	154 (28.6)	1.45 (0.91-2.32)	0.131	483 (89.6)	1.15 (1.02-1.31)	0.004
Frequency of ANC attendance (last pregnancy)										
<4 times	140	13 (9.3)	1.0		41 (29.3)	1.0		122 (87.1)	1.0	
4 or more	478	59 (12.4)	1.04 (0.97-1.10)	0.371	129 (27.0)	0.89 (0.59-1.35)	0.667	423 (88.5)	1.14 (0.64-2.01)	0.766
Place of delivery of the last child										
Home delivery	31	1 (3.2)	1.0		9 (29.0)	1.0		28 (90.3)	1.0	
Facility delivery	587	71 (12.1)	4.14 (0.56-30.79)	0.16	161 (27.4)	0.95 (0.54-1.66)	0.838	517 (88.1)	0.98 (0.87-1.09)	0.789
Ever heard of rubella										
No	546				140 (25.7)	1.0		475 (87.2)	1.0	
Yes	72				30 (41.7)	1.62 (1.19-2.21)	0.005	69 (95.8)	1.10 (1.04-1.17)	0.03
Mention syphilis as an MTCT infection										
No	545	140 (25.7)	1.0					379 (84.6)	1.0	
Yes	172	30 (41.7)	1.62 (1.19-2.21)	0.005				166 (97.6)	7.56 (2.71–21.04)	<0.001
Mention syphilis as an MTCT infection										
No	68	475 (87.2)	1.0		379 (84.6)	1.0				
Yes	550	69 (95.8)	1.10 (1.04–1.17)	0.03	166 (97.6)	7.56 (2.71–21.04)	<0.001			

**Table 5:** Multivariate analysis of factors associated with rubella, syphilis, and HIV as MTCT infections.

Variable	Rubella		Syphilis		HIV	
	Adjusted OR (95% CI)	*P* value	Adjusted OR (95% CI)	*P* value	Adjusted OR (95% CI)	*P* value
District of enrollment						
Rombo	1				1	
Moshi urban	1.28 (0.63-2.61)	0.499			0.37 (0.19-0.75)	0.005
Same	2.15 (1.18-3.92)	0.012			0.49 (0.25-0.94)	0.032
Age group (years)						
15-24			1		1	
25-34			1.67 (1.02-2.75)	0.04	1.44 (0.77-2.69)	0.255
35+			2.99 (1.76-5.11)	<0.001	0.83 (0.42-1.64)	0.588
Marital status						
Married/cohabiting			1.0		1.0	
Single			0.78 (0.44-1.38)	0.393	0.50 (0.26-0.95)	0.034
Widow/divorced/separated			1.04 (0.49-2.25)	0.912	2.31 (0.51-10.40)	0.275
Women's occupation						
Not formally employed			1.0			
Formally employed			2.90 (1.35-6.23)	0.006		
Have mobile phone						
No					1.0	
Yes					2.29 (1.21-4.36)	0.011
Ever heard of rubella						
No			1.0		1.0	
Yes			1.99 (1.17-3.40)	0.011	3.66 (1.08-12.36)	0.037
Mention syphilis as an MTCT infection						
No	1.0				1.0	
Yes	1.93 (1.15-3.23)	0.013			6.89 (2.43-19.56)	<0.001
Mention HIV as an MTCT infection						
No	1.0		1.0			
Yes	2.97 (0.89-9.85)	0.08	6.96 (2.47-19.63)	<0.001		

## Data Availability

The datasets used and/or analyzed during the current study are available from the corresponding author on reasonable request.
